# A Long Case

**Published:** 1888-10-13

**Authors:** 


### A LONG CASE.

"As showing the contingencies that frequently arise in hospitals," says Dr. W. McEwan, "I remember the case of a grown-up girl, who came to the hospital (the Glasgow Royal Infirmary) so severely burned that there was not a particle of skin on her back from the neck to the hip. The late Dr. Watson, with a perseverance which did him credit, patched the whole back with little pieces of skin daily, taken from other parts of the body, and in some cases contributed by other patients who willingly offered themselves. This cure took about fifteen months to accomplish, but it was a complete cure, and the girl went out of the hospital hale and hearty."

